# The Acceptance, Usability, and Utility of a Web Portal for Back Pain as Recommended by Primary Care Physicians: Qualitative Interview Study With Patients

**DOI:** 10.2196/38748

**Published:** 2022-12-29

**Authors:** Christian Schlett, Nicole Röttele, Piet van der Keylen, Andrea Christina Schöpf-Lazzarino, Miriam Klimmek, Mirjam Körner, Kathrin Schnitzius, Sebastian Voigt-Radloff, Andy Maun, Mario Sofroniou, Erik Farin-Glattacker

**Affiliations:** 1 Section of Health Care Research and Rehabilitation Research Medical Center – University of Freiburg, Faculty of Medicine University of Freiburg Freiburg Germany; 2 Institute of Medical Psychology and Medical Sociology Faculty of Medicine University of Freiburg Freiburg Germany; 3 Institute of General Practice University Hospital Erlangen Friedrich-Alexander University Erlangen-Nürnberg Erlangen Germany; 4 Institute for Evidence in Medicine Faculty of Medicine and Medical Center University of Freiburg Freiburg Germany; 5 Institute of General Practice / Family Medicine Medical Center – University of Freiburg, Faculty of Medicine University of Freiburg Freiburg Germany

**Keywords:** general practice, primary care, lower back pain, digital health intervention, web-based health information, eHealth, patient education, adherence, qualitative research, framework analysis, mobile phone

## Abstract

**Background:**

An ever-increasing number of patients seek health information via the internet. However, there is an overabundance of differing, often low-quality information available, while a lack of health literacy makes it difficult for patients to understand and assess the quality and trustworthiness of the information at hand. The web portal tala-med was thus conceived as an evidence-based, up-to-date, and trustworthy information resource for lower back pain (LBP), which could be used by primary care physicians (PCPs) and patients during and following consultations for LBP. The current evidence demonstrates that patients with LBP could benefit from web portals. However, the use of such portals by patients remains low, thus limiting their effectiveness. Therefore, it is important to explore the factors that promote or hinder the use of web portals and investigate how patients perceive their usability and utility.

**Objective:**

In this study, we investigated the acceptance, usability, and utility of the web portal tala-med from the patient perspective.

**Methods:**

This qualitative study was based on telephone interviews with patients who had access to the web portal tala-med from their PCP. We used a semistructured interview guide that consisted of questions about the consultation in which patients were introduced to tala-med, in addition to questions regarding patient perceptions, experiences, and utilization of tala-med. The interviews were recorded, transcribed, and analyzed through framework analysis.

**Results:**

A total of 32 half-hour interviews were conducted with 16 female and 16 male patients with LBP. We identified 5 themes of interest: the use of tala-med by PCPs during the consultation, the use of tala-med by patients, its usability, added values derived from its use, and the resultant effects of using tala-med. PCPs used tala-med as an additional information resource for their patients and recommended the exercises. The patients appreciated these exercises and were willing to use tala-med at home. We also identified factors that promoted or hindered the use of tala-med by patients. Most patients rated tala-med positively and considered it a clear, comprehensible, trustworthy, and practical resource. In particular, the trustworthiness of tala-med was seen as an advantage over other information resources. The possibilities offered by tala-med to recap and reflect on the contents of consultations in a time-flexible and independent manner was perceived as an added value to the PCP consultation.

**Conclusions:**

Tala-med was well accepted by patients and appeared to be well suited to being used as an add-on to PCP consultations. Patient perception also supports its usability and utility. Tala-med may therefore enrich consultations and assist patients who would otherwise be unable to find good-quality web-based health information on LBP. In addition, our findings support the future development of digital health platforms and their successful use as a supplement to PCP consultations.

**International Registered Report Identifier (IRRID):**

RR2-10.1186/s12875-019-0925-8

## Introduction

### Background

An ever-increasing number of patients access health information via the internet [1-3]. However, most of the health-related information available on the web is of low quality, while many patients are unable to adequately appraise the quality of such health information [4,5]. *Tala-med* was therefore envisaged and developed as an evidence-based, up-to-date, and easily understood web-based information resource for lower back pain (LBP) through the *Gut informierte Kommunikation zwischen Arzt und Patient* (GAP) project (well-informed communication between the general practitioner and patient) [6]. LBP in particular is a widespread health problem that causes substantial personal and financial burden [7,8]. In Germany, LBP has a 1-year prevalence of more than 60% [9,10], accounts for the most days of sick leave (6.1%) [11], and is one of the most common reasons why people visit their doctor [12].

Despite the existence of national and international clinical guidelines, approaches to treating LBP differ greatly among clinicians, institutions, and geographic regions [13,14]. However, the breadth of available web-based information often surpasses the variety of management approaches. Consequently, patients can often be confused and frustrated while searching for web-based information regarding LBP [15]. An overabundance of differing and contradictory information can make it difficult for patients to understand and assess the quality and trustworthiness of the information provided [15], often presenting a dilemma for patients searching web-based information for LBP.

Digital health interventions (DHIs) recommended by health care professionals (HCPs) may be a remedy for this dilemma, as they can provide patients with tailor-made, understandable, and high-quality information. Our web portal *tala-med* is one such DHI that could be recommended to patients by primary care physicians (PCPs). As an information resource based on national [16,17] and international clinical guidelines for LBP [18-20], *tala-med* can be classified as a DHI that can provide health content [21,22] to physicians and patients alike. *Tala-med* aims to improve the shared decision-making of PCPs, while enhancing patient-informed choices, participation, and self-management regarding LBP [6]. For interventions on shared decision-making to be most effective, Cochrane reviews have shown that these should be both physician and patient focused and include information that is indication specific [23,24]. Regarding DHIs on back pain, recent meta-analyses and systematic reviews have shown that such interventions, especially those that focus on self-management, can have clinically important effects in terms of relieving patient discomfort and improving their disability [25-27]. However, a key determinant of the effectiveness of DHIs on LBP [28] and DHIs in general [29] is adherence; that is, whether patients actually use the DHI to the intended extent [30].

Unfortunately, the extent to which patients use DHIs is often low [31-33]. In addition, promoting the use of DHIs by patients is complex, with only limited evidence available on successful strategies to do so [29]. Therefore, it is important to understand the web-based health information needs of patients [34] and other factors that may facilitate or hinder their use of a DHI. Regarding web-based information on LBP received by patients as an adjunct to their PCP consultation, Riis et al [15] found that readability, customization, design, credibility, and usability are important domains. However, these results were based on patient experiences using a variety of health-related websites and not on the use of a specific web portal provided by their PCP. Studies on patient perceptions of DHIs for LBP have also revealed that contextual factors, such as the support of HCPs, and individual factors, such as patient skill and preference, affect the acceptance of such DHIs [28,35]. A current systematic review of qualitative studies found only 4 studies that investigated the facilitators of and barriers to the use of DHIs by patients with LBP. Svendsen et al [28] thus state that “further primary research investigating the implementation of DHIs and user’s experiences is required.”

### Objectives

The aim of this study was to examine patient acceptance of our web portal *tala-med* as well as its usability and utility. We were eager to see how PCPs used the portal during the consultation, how they offered it to patients, and whether it was subsequently used. We also wanted to understand which parts of the portal were considered helpful and how patients perceived *tala-med* in terms of key characteristics such as comprehensibility and trustworthiness, enabling us to identify the portal’s strengths and weaknesses*.* This in turn provided us with insights into how specific features of the portal or its setting may have contributed to patient perception. Finally, we examined the utility of *tala-med*, in particular, the perception of any added value brought about by its use.

## Methods

### The Web Portal Tala-med

**Figure 1:**
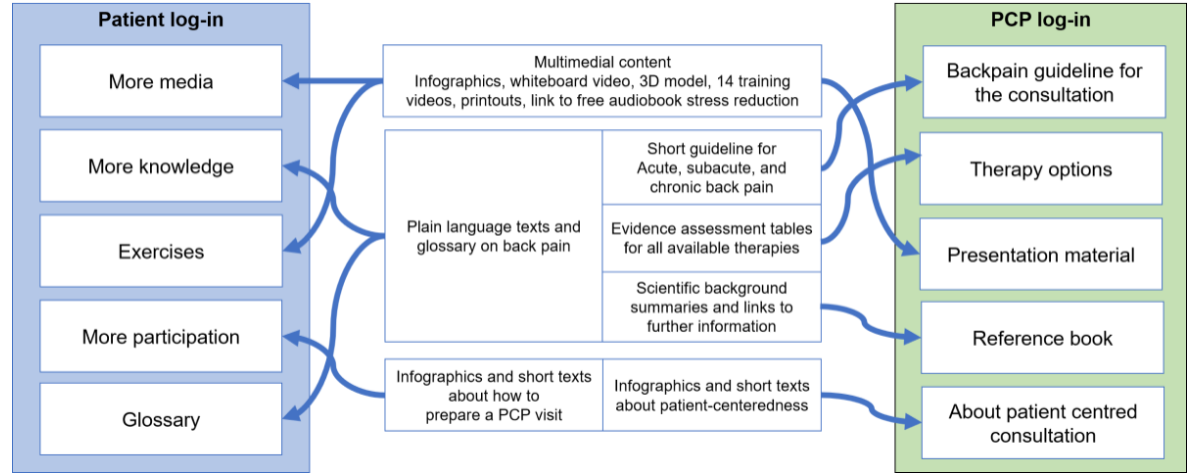
Elements of the web portal tala-med. PCP: primary care physician.

*Tala-med* is a German-language, evidence-based, comprehensible, and reliable internet information portal for LBP that is freely accessible and aimed at improving patient informedness and patient-doctor interaction. It was implemented within a prospective cluster-randomized controlled trial (RCT) with pre–, post– and 1 follow-up measurement [6]. *Tala-med* was designed to be used by PCPs and patients in the intervention group during and after LBP consultations. There are 2 versions that have been adapted to meet the demands and linguistic levels of PCPs and patients (Figure 1). To log in to the portal, both PCPs and patients were given an individual fictitious username consisting of a combination of 2 short animal, color, and fruit words (eg, PearOwl) and a password. This type of log-in was necessary because portal use data were also analyzed anonymously as part of the RCT [6].

The portal was developed over a 6-month period by a multidisciplinary team consisting of 1 PCP, 2 physiotherapists, 2 researchers specializing in evidence synthesis, 2 web designers, a film crew concerned with exercise video production, a specialist for whiteboard videos, and a 3D specialist. The focus was on frequently occurring issues seen in LBP, and it contained descriptive material in short and long versions, such as an accordion style guideline, where users could click on headings and highlighted keywords to obtain further background information. The development of the portal, including information processing and design principles on which the presentation of the information was based, is described in the associated methodological guide, which is also publicly available [36]. The final version consisted of 6 infographics, with 20 illustrations, a 3D model animation of the lower back, an 11-minute explanatory whiteboard video containing 8 different topics, and 14 short exercise videos 2 to 4 minutes long, with an emphasis on teaching self-care. In addition, suggestions, infographics, and background information on well-informed shared decision-making and preventive lifestyle changes were also included. The PCPs were able to show or print material during their consultations. Study PCPs were instructed to show and explain the web portal to their patients and encourage its use. If this was not possible, for instance, if the consulting room had no computer access, PCPs were advised to promote its use, despite being unable to demonstrate the portal in real time. As a backup option, PCPs were given up to 4 hard-copy brochures, which could be handed out to patients who were unfamiliar with computer use. The restriction to 4 brochures was chosen to encourage PCPs to primarily use the web portal and only refer to a brochure when handing the portal to a patient seemed inappropriate. The brochures contained information from the web portal in printed form. Patients were able to use the patient-tailored version of the web portal at home.

### Study Design

We used a qualitative design in which patients with LBP, who had received access to *tala-med* via their PCP, were invited by post to individual telephone interviews. For administrative reasons and owing to the delay of patient postal responses, interviews were held 1 to 2 months after the consultation, in which patients received their log-in details to the web portal. Results were reported according to the consolidated criteria for reporting qualitative research (consolidated criteria for reporting qualitative research [37]).

### Recruitment

All 190 patients with LBP from the intervention group were invited to participate in an interview after the last follow-up measurement of the RCT. Although patients received a book voucher for their participation in the RCT, no incentives were offered for their participation in the subsequent interview study. Then, 35 patients (18%) accepted the invitation and returned their informed consent and contact information. Three patients were not interviewed: 2 could not be reached and 1 was no longer interested in an interview, as she had not yet used the web portal. A total of 32 patients were interviewed, and 17% of all invitees were interviewed. The patient characteristics are shown in Table 1.

**Table 1:** Patient characteristics (N=32).

Characteristic		Patients, n (%)
**Gender**		
	Male	16 (50)
	Female	16 (50)
**Age (years)**		
	18-29	1 (3)
	30-39	8 (25)
	40-49	4 (13)
	50-59	10 (31)
	60-69	8 (25)
	70-79	1 (3)
**Level of education (highest level completed)**		
	Elementary school	10 (31)
	Secondary school	15 (47)
	High school diploma	7 (22)
**Do you currently still have back pain?**		
	Yes	18 (56)
	No, not at the moment	7 (22)
	No (without any remark)	7 (22)
**First experience of back pain in your life?**		
	<6 weeks	0 (0)
	6-12 weeks	1 (3)
	>12 weeks to <1 year	0 (0)
	1 to <2 years	2 (6)
	2 to <5 years	3 (9)
	5 to <10 years	3 (9)
	>10 years	23 (72)

### Ethics Approval

The study was approved by the ethics committee of the Albert-Ludwigs-University Freiburg (no 559-17).

### Informed Consent

All participants provided written informed consent before being interviewed. Apart from contact data, the interviewers had no prior information about the participants. At the beginning of the telephone interviews, patients were informed about data protection issues and the recording of the interviews (Multimedia Appendix 1).

### Data Collection

Interviews were held between February 2019 and October 2020 by CS (31 interviews) and M Klimmek (1 interview). These were based on a semistructured interview guide developed by ACSL, CS, and NR, which contained questions about patient experiences of the consultation and web portal (Multimedia Appendix 1). The interview guide contained skip rules to ensure that interviewees were only asked questions that they could provide answers for. For example, patients who did not use the portal were not asked questions regarding the usability of the portal and its utility. The interviews lasted an average of 30 minutes, ranging from 15 to 45 minutes in length. Interviewers did not take field notes during the interviews. The interviews were digitally recorded by connecting a digital recorder to the interviewer’s landline telephone. Recordings were transcribed verbatim by a transcription service and analyzed using MAXQDA (version 20; VERBI Software GmbH). Participants did not receive the transcripts or feedback results.

### Data Analysis

The transcribed interviews were analyzed using framework analysis [38-40]. Textbox 1 outlines the 5 stages of the analysis as well as their implementation.

**Stages of the framework analysis.**

- Familiarization: the purpose of this stage was to become immersed in the data to gain an insight into its range and diversity [39]. Therefore, CS read the transcripts of 10 in-depth and heterogeneous interviews.
- Identifying a thematic framework: this stage was concerned with developing a code system that covered the most important issues [39]. Codes were first developed deductively based on the main themes of the interview guide. They were then expanded inductively with codes covering the emerging issues. In this vein, CS coded 10 transcripts and created a code system. Because a single look at the data might miss important issues or overemphasize less important ones, we included a second view. Therefore, NR coded 5 transcripts independently and also created a code system; CS and NR discussed and combined their code systems into one. With this new code system, CS coded the next 14 transcripts and refined the system accordingly. To verify the refined system, 3 in-depth and heterogeneous transcripts (of the 14 last coded ones) were coded again independently by NR with the refined code system. CS and NR compared the coding of these 3 interviews. Because of high consistency, only a few changes were necessary to create a final code system.
- Indexing: this stage described the application of the code system to the entire set of data [39]. CS and M Klimmek applied the final code system to all transcripts and verified its application reciprocally.
- Charting: at this stage, coding was extracted and tabulated, with columns representing codes and rows representing patients. This allowed codes to be read horizontally for a given patient or patients to be read vertically for a given code. CS and M Klimmek condensed and summarized the coding as far as possible in the patients’ own words and created a chart with codes represented as columns and patients as rows.
- Mapping and interpretation: this stage concerned the mapping out of the data and making sense of it [40]. It may include a description of the range and nature of phenomena and searching for associations between and within codes to find explanations for the research questions [39]. To lay out and make sense of the data, we used a method called one sheet of paper (OSOP) [41]. This method entailed reading the condensed extracts of each code and summarizing all the different issues of a code on OSOP. This summary of the different issues was then used as a basis for axial coding, that is, for considering which issues group into broader themes and to “develop an explanation of ‘what is going on in the data’ that takes account of all the issues raised” [41]. In this way, CS summarized the different issues of each code, oversaw the summary of issues, and considered how they form broader themes that could provide explanations for the research questions. Five core themes emerged from this overview: primary care physician (PCP) use of the portal during the consultation, patient use of the portal, usability, added value, and effects of the portal. Regarding these themes, CS selected the codes that provided information (Multimedia Appendix 2) and searched for associations between the codes of each theme and between the themes. CS made a first draft of results and discussed and refined it with M Klimmek. The refined results were discussed with NR, PK, ACSL, M Klimmek, M Körner, SVR, and EFG and adapted by CS.

As described in Textbox 1 and in the previous section, CS, ACSL, M Klimmek, and NR were the researchers primarily involved in data collection and analysis. CS and ACSL were postdoctoral researchers in the field of health services research and rehabilitation research, who hold degrees in psychology. M Klimmek was a bachelor’s student in social work who worked as a student assistant in the same field. NR is a researcher in the field of medical psychology and medical sociology who holds a degree in health education. ACSL is experienced with qualitative studies. She introduced CS and NR to the framework analysis.

## Results

We identified 5 core themes using framework analysis: PCP use of the portal during the consultation (theme 1), patient use of the portal (theme 2), usability (theme 3), added value (theme 4), and effects of the portal (theme 5).

### Acceptance

Two themes were related to patient acceptance of the portal: PCP use of the portal during consultation (theme 1) and patient use of the portal (theme 2).

#### PCP Use of the Portal During Consultation

This theme reflected on how PCPs introduced the portal to patients and the usage they encouraged. The following represents patient perceptions of PCP behavior during consultation. All 32 patients interviewed provided insight into their perception of the consultation, although 9 of these patients also expressed difficulty in recalling the consultation, as it had taken place so long ago. The degree to which PCPs introduced the portal to their patients varied greatly: from detailed explanations with a demonstration to some explanation to no explanation at all. Many patients reported that their PCP had explained and showed them the portal, either on the screen or by using the brochure. Some also received printouts of exercises or other information from the portal of their PCP. Others reported that their PCP described aspects of the portal, such as the log-in, use, and contents, without showing it. These patients received, as a minimum, general information or a recommendation, for instance, “the portal contains exercises that might be helpful to you” (patient 3, male). Some patients were simply asked to participate in the study or were given log-in details by a physician assistant without any further information.

The PCPs who mentioned the portal mainly suggested that the patients used the exercises. Other aspects of the portal, for instance, those aimed at knowledge transfer or patient-doctor communication, were suggested to only a few patients. Even if PCPs showed their patients the portal, most patients perceived the consultation as per usual or differing only slightly from previous consultations. Only a few patients reported any noticeable changes in the consultation owing to the use of the portal, such as more in-depth and informative conversations, or more time and interest on the part of their PCP. Patient satisfaction with the consultation seemed unaffected by PCP use of the portal. Rather, it depended on the general aspects of the consultation, such as how much time the PCP devoted to them, whether the conversation was perceived as in depth, whether the PCP seemed interested in their recovery, or whether patients received the treatment they had hoped for. Most patients whose PCP used the portal endorsed the use of the portal in future consultations. However, 1 patient expressed concerns about how the portal should be used:

Only if the PCP uses it in detail, so that it’s not such an everyday project.

Patient 22, female

I beg your pardon? Only if the PCP?

Interviewer

Well, if the PCP really works intensively with it, so that he doesn’t just say: hey, there’s a program, do this and bye-bye, handing over another piece of paper, but really goes into it in more detail.

Patient 22, female

#### Patient Use of the Portal

Of the 32 patients interviewed, 4 used both the web portal and the brochure, 22 used the portal alone, 2 used the brochure alone, and 4 used neither the portal nor the brochure. If a patient used the portal, the interview focused on its use, even if the patient also used the brochure.

Textbox 2 presents the facilitators of and barriers to portal usage. As in previous studies, factors relating to the initial use of the portal did not differ from those relating to the continued use of the portal [28] and were therefore reported together. Seeing the portal during the consultation raised initial patient interest and shortened the time to their first log-in.

**Facilitators of and barriers to portal usage (N=32).**

**Facilitators**

- Conditions and treatments
  Primary care physicians (PCPs) showed portal in the consultation
  PCPs recommended exercises
  Back pain started again
  Comorbidity that benefits from exercise
- Organization and motivation
  Time-flexible use of the portal
  Emerging specific questions
  High self-motivation of patients

**Barriers**

- Conditions and treatments
  Currently absent or severe back pain
  Other more intensive back pain therapies
  Other health problems had priority
- Organization and motivation
  Lack of time (due to work, household, childcare, or care for older adults)
  Lack of motivation, patience, or concentration
  Portal provided no new suggestions
- Technical requirements and skills
  PC or internet problems
  Lack of PC skills
  Use on smartphone not possible
  Log-in: details lost or did not work

When asked about when he first looked at the portal, a patient who had been shown the web portal during the consultation replied as follows:

Well, it was either on the same day or the day after, just out of curiosity.

Patient 5, male

So, you logged in straight away and looked at what was available?

Interviewer

Exactly. I read it through more carefully myself to see what exercises there are and...Yes, it was actually a good suggestion by the physician.

Patient 5, male

In addition, patients were more willing to try exercises from the web portal if their PCP recommended them. They were also more likely to use the portal if they had a recurrence of LBP. No LBP and too much LBP were barriers to portal use: patients saw no reason to use *tala-med* if they no longer had any LBP or equally were unable to use it if they were experiencing excessive LBP. Similarly, patients did not use the portal if they underwent more intensive treatment for LBP, such as in-patient therapy. Some patients were unable to use the portal because of other acute conditions, especially mental health conditions, such as depression. However, if they had another condition that also benefited from exercise, such as hypertension, they were more willing to use it.

The nature of the portal as a time-flexible web-based tool made it easier for patients to fit its use in their daily routines. Patients were inclined to use *tala-med* when new questions about LBP arose, as well as when they demonstrated increased motivation to improve their own health. Lack of time owing to other commitments was a frequent barrier to their use. A lack of motivation, patience, or concentration was a reason why patients used the portal less often or no longer. Patients were also abstained from further use if new suggestions within the portal could not be found. Technical requirements and skills were also important barriers to their use. If a patient’s device or internet connection did not work or they were unfamiliar with their functions, they also, understandably, did not use the portal. Some patients held reservations about using the portal owing to difficulties using a computer or the internet. Lost log-in details or other log-in difficulties were also obvious barriers to patient use.

Most of the 26 patients who used the portal logged into it for the first time within the first 3 days following a consultation. The exercises were most often perceived as the most helpful part of the portal. A substantial proportion of patients were interested in web portal exercises to alleviate their pain but showed no interest in other aspects of the portal:

This theoretical background did not interest me further in this case. I just wanted to do these exercises.

Patient 5, male

I was really only interested in the exercises, because they...well...help the most and, no, I didn’t want to look up or know more.

Patient 16, female

Nonetheless, the other 3 sections—more media, more knowledge, and more participation (Figure 1)—were also perceived by some of the patients as being the most helpful parts of the portal.

### Usability

Responses regarding the usability of the portal (theme 3) relied on the answers of 26 patients. However, 10 of these patients mentioned that they had difficulty remembering the portal in detail. Textbox 3 shows how patients rated the portal and their respective reasons. In general, most patients rated the portal as positive. They mentioned that the portal was interesting and informative, provided good information, was easy to use and easy to implement, and contained useful exercises and videos. Neutral and negative overall ratings were obtained from patients who were disappointed that the portal did not recommend their preferred treatment (injection) or lacked information about a particular type of back pain (upper back pain).

Reasons for positive design ratings were that the portal was perceived as visually well-structured and uncluttered. Patients also liked the brevity and simplicity of the exercise videos:

I also find the videos very beautiful. Not so crowded, but just a person who shows this, does that. Not so much jumping around and stuff, like some others there, when you look on the internet and everyone thinks, they have to do fancy other things, but very simple. You do that, that’s how it should be done, and then you do it that way and that’s it. So, I think it’s good just as it is.

Patient 30, male

Patients with neutral or negative perceptions of the portal design reported that they preferred dealing with a real person, needed more interactive elements and animations, and found that the portal was poorly designed for smartphone use. The portal was optimized for notepad-sized screens or larger screens.

Most patients perceived the portal as clear and did not have any problems interacting with it. They found it simple and well structured, well described, and thus easy to navigate. For some patients, the portal structure was unclear; 1 patient stated that he could not find everything he needed from the outset, reporting that “it would be easier for a younger person who sits in front of the computer all the time” (patient 21, male, age category: 50-59 years). The other patients had problems dealing with the portal, needing help, or finding it too convoluted and deeply structured, requiring too many clicks to find what they were searching for.

Almost all patients perceived the portal information as easy to understand, even for back pain novices, because the information was rated as simple and well described, containing only a few specific terms with no extensive texts. The portal information was unanimously perceived as trustworthy. Patients mentioned 4 main reasons for this (Textbox 4). Many patients perceived the portal as trustworthy, as it was developed or recommended by a source with a high level of expertise:

How trustworthy did you find the information on the platform?

Interviewer

Very trustworthy.

Patient 32, male

On what did you base that on, the trustworthiness?

Interviewer

I put it down to the fact that my doctor recommended it to me and I actually trust her very much.

Patient 32, male

**Overall and design ratings of the web portal (N=26).**

**Overall rating**

- Positive
  Good information
  Well-explained exercises, easy to implement
  Very good background and explanations
  In sum very interesting, very informative
  Quite good at the beginning for browsing
  Pragmatic and practicable
  Very easy to use, well structured
  Simplified, everyone will get along well with it
  Great videos
- Neutral
  The portal did not recommend patients’ preferred treatment
  I can’t judge it
- Negative
  Too little info about upper back pain

**Design**

- Positive
  Yes and no and questions and answers is good
  Very appealing, not too cluttered
  Videos are great
    - From the duration
    - Not so much jumping around
    - Super to see a normal person (no super athlete)
  Visually well constructed
  Nothing visually disturbing
- Neutral
  No real person behind it
  Not amazing but not bad either
  I did not despair of it
- Negative
  Conservative and classic, too little interactive and animated
  Very smartphone unfriendly

**Clarity, comprehensibility, and trustworthiness of the web portal (N=26).**

**Clear**

- Yes
  I had no problems finding my way around
  Very simple overview, for the inexperienced
  You quickly get to where you want to be
  It was easy to find specific information
  Everything is well described and clear
  Well structured
- Partly
  It took a bit to get into it, but then it was clear
- No
  I didn't get on with it, needed help
  Easier for a younger person, who always sits in front of the computer
  Too convoluted, too deeply structured
  I have to click a lot until I find what I am looking for

**Comprehensible**

- Yes
  Everything is very simple and well described
  Few strange words or specific terms
  No long texts
  Many things are relatively well explained
  Easy to understand even for back pain novices
  Videos are very well described, they are self-explanatory
- No
  Contained technical terms, which the patient did not comprehend

**Trustworthy**

- Yes
  Trustworthy source with high expertise
    - Recommended by primary care physician (PCP)
    - Developed by experts (doctors or universities)
  Well-founded, scientific info
    - Well-founded impression
    - Very informative and scientifically developed
    - Emphasis on info, not on fuss
  Accurate, noncontradictory information
    - Things patient knows to be true
    - Nothing contradictory
  Serious presentation
    - Not the impression of advertising
    - Much attention to data protection
    - Fictitious username and password
  Positive intention noticeable

Furthermore, underpinnings of trustworthiness were the well-founded and scientific information of the portal and its serious presentation, putting an emphasis on the clear presentation of information, while avoiding distractions or advertisements. Patients also perceived the portal as trustworthy, as it contained noncontradictory information with aspects the patients knew to be true. The use of a fictitious username with great attention to data protection also supported the impression of trustworthiness for some patients.

A total of 14 patients had suggestions for improvement. Regarding additional content, patients suggested adding the addresses of recommended specialist centers, doctors, or therapists in their region, with freely available consultation slots when a second opinion was needed at short notice, as well as an advice hotline on the contents of the portal. Including more exercises for the upper back, alternative therapies such as acupuncture, and offering support for other conditions in addition to LBP were also suggested. Design improvements were also envisaged through the use of greater customization, with separate access for patients with little experience of LBP, as well as with more experience of LBP. Regarding the exercise videos, shorter sequences for use on the go, as opposed to the current 2- to 4-minute videos on offer, would have been appreciated, as well as the ability to loop videos, adjusting the number of repetitions available for exercising in tandem with the videos. One patient would have appreciated instructions to be given throughout the program, while another patient felt the need for a diagnosis-orientated search facility, with diagnosis-specific preselection of information and exercises. Regarding accessibility, many patients felt that the portal should be made freely available to everyone, which was unfortunately not the case at the time of the study. One patient suggested that the portal be made widely available in waiting rooms and pharmacies. Some hoped that it would be made available as a smartphone app, while others were eager for a hardcopy printout to be made available to those without a computer or access to the internet. Almost all patients affirmed that they would recommend the portal to others and some had already done so.

### Utility

The utility of the portal was assessed by its added value (theme 4) and its effects (theme 5).

#### Added Value

If patients are offered a web portal by their PCP as a supplement to the consultation, this should bring added value to the consultation or to preexisting sources of information that patients may otherwise have access to. Patients highlighted the trustworthiness and validity of the information available through *tala-med* as bringing added value above and beyond those gained by accessing other sources of information, such as self-guided internet searches (Textbox 5).

Patients also praised the comprehensiveness of the information provided in *tala-med* and its appropriateness. In addition to the perceived value of such features, patients appreciate the time and effort saved when searching for health information. Patients also see added value in watching the exercise videos, as opposed to paper instructions or reading them on the web, which may be less easy to understand. In addition to these specific aspects of the portal, the practical relevance and the positive effects on LBP were also seen as bringing added value:

The added value for me was definitely that it comes from a clinic and my doctor recommended that I should use it. That was actually the added value for me and that I can draw the conclusion that I definitely feel better because of it.

Patient 32, male

Two patients saw no added value in using *tala-med* as they felt it did not offer any new suggestions or corrections to existing exercises, as one would experience in a face-to-face course, the latter being preferred by one of the patients.

Compared with a PCP consultation without subsequent access to *tala-med*, patients saw added value in the possibility to repeat, deepen, and reflect on the contents of the consultation (Textbox 6).

How much (given as a percentage) does one really remember in a doctor-patient conversation? Not that much, right? And then you can just read about it [in the portal]. And that was quite good [...] You can just have another look: Ah...Now I have another question. Or [I] can take another look: Now that would have interested me, I forgot [that]. See if I can find something in there. Well, I find it good as an additional offer. Of course, it doesn’t replace the personal doctor-patient consultation.

Patient 15, female

Patients appreciated the time-flexible and independent use of the portal, which also supported their active role in taking care of their own LBP. Having access to the portal motivated patients to engage with the content and made it easier for them to implement the necessary exercises. Patients also saw the positive effects of using the portal (see the *Effects* section) as bringing added value to the PCP consultation. They stated that less would have been known about their LBP and less exercise would have been done had they not had access to *tala-med*.

**Added value of the web portal to other sources of information (N=26).**

**Trustworthiness**

- Better than surfing and only coming across advertising or nonsense content
- The content and motives of the portal do not need to be verified, as the portal is trusted
- One knows that one is on the right website

**Information validity**

- Content is valid, more profound, well founded, and professional than what is found elsewhere on the internet
- Portal contains independent information; it contains more than just one person’s experience

**Comprehensive information**

- Very bundled and compact; it comprises causes, treatment options, and exercises all in one
- Saves time-consuming search on the internet or elsewhere; other sources of information become unnecessary

**Appropriate information**

- Information is prescreened, specific to back pain (DVDs with exercises are often less specific)
- Suits what the patient is currently dealing with
- Facilitates further searches for appropriate information

**Good exercise videos**

- Exercise videos are better and more motivating than instructions on paper
- Exercises are well explained, which was not the case with the results of an internet search

**Added value of the web portal to the primary care physician (PCP) consultation (N=26).**

**Additional information to the PCP consultation**

- To repeat:
  To be able to read at leisure again or recall what the PCP said
  No need to remember everything from the conversation because patients could read it again later
  Reading up later removes uncertainty
- To deepen:
  To deepen what the PCP said; provided targeted additional information beyond the normal consultation
  Supplementary knowledge and new exercises
- To reflect:
  Allows comparison with what was said by the PCP
  Be better informed to ask my PCP questions next time

**Time-flexible use**

- Visit the website whenever I wanted and when I had time; always accessible
- Glance quickly when I have a pain episode; start directly with exercises, do not have to wait for an appointment

**Independent use**

- Practical solution for home, I could do something without needing a health professional
- Can be flexibly integrated into my daily routine, one is not dependent on someone else

**Motivates engagement**

- Pushes me to move more; encourages me to overcome my weaker self
- Compulsion to do something until the next consultation (to familiarize myself, to try something out)

**Facilitates implementation**

- Exercises are well explained and easy to follow because of the videos

#### Effects

Overwhelmingly positive outcomes were mentioned by patients, highlighting the effects that using the portal had on their degree of LBP, informedness, and patient participation. Many patients were able to alleviate their LBP by using the portal, specifically as a consequence of doing the exercises. Unfortunately, 1 patient with preexisting severe LBP experienced worsening LBP while performing exercises. Patients perceived the portal as an impetus to do more exercise regularly, as well as more sport or movement in general. They also felt that doing the exercises might decrease or delay their need to visit their PCP again.

Using the portal also increased the informedness of patients with LBP. It provided them with an overview of treatments, improving their understanding of LBP, as well as its causes, while offering new perspectives on their own contribution to their LBP. Increased informedness and shared information with their PCP were seen as advantageous for upcoming consultations. Patients felt that they could (1) enter consultations with better prior knowledge, (2) ask targeted questions more easily, and (3) speak to their PCP about specific topics and exercises. Patients also assumed that increased informedness might reduce their need for PCP visits:

Do you think that...if you use the portal, it influences conversations with your PCP or with other people in the healthcare system?

Interviewer

Yes, definitely! Definitely. Because, after all, there are many people who don’t know where back pain comes from. Or how to avoid it. I actually think that maybe you don’t need the doctor as much.

Patient 18, female

Using the portal also facilitated patient participation, increasing patient awareness of the importance of the interaction with their PCP and encouraging them to express their thoughts and concerns through greater participation:

I have become more open in conversations with the doctor, so that I have dared more or have understood that I actually have to say what I think, only then can we really talk about it...it became clear to me that it is also from my side, yes...that I also have thoughts about my illness, or about my pain, and that I don’t just have to perceive as valid what the doctor tells me.

Patient 11, male

For the next PCP visit, I’ll write down a few questions [in advance] and I’ll be more pushy about my problems or my wishes.

Patient 8, male

## Discussion

### Principal Findings and Comparison With Prior Work

Patients accepted the portal well, appreciating its use both in the consultation and at home. PCPs mainly used the portal as an additional information resource for their patients and recommended the exercises as described. Although patients appreciated this use, it only partially exploited the information potential of the portal. The parts of *tala-med*, which aimed to improve the consultation and in particular shared decision-making, seem to have had little impact. With respect to these aspects, the findings suggest that *tala-med* or its implementation could be improved. Many patients perceived the exercises, presented as guided videos, as the most helpful part of the portal, this being the sole area of interest for some. This finding supports the suggestion of Wollmann et al [34] that videos and tutorials about health information would be well received by patients.

Patient use of the portal was facilitated through PCP behavior during the consultation, such as introducing the portal and recommending it to patients. This finding is consistent with previous studies showing that HCP recommendations and support for the DHI were facilitators of use [28]. The current evidence underlines the important role of HCPs in promoting patient use of DHIs. Our findings also showed that portal use was facilitated through further questions from patients and through new onset and moderate LBP, which also served as a reminder to patients to take action. Other levels of LBP, in particular no LBP or excessive LBP, hindered patient use of the portal. According to a current systematic review, DHIs for the self-management of LBP should be tailored to pain severity [28]; otherwise, patients would not use them [15,28,42]. Our finding that absent or severe LBP was a barrier to portal use may reflect the degree to which *tala-med*’s contents align with pain severity, suiting patients with mild to moderate pain but less so those with absent or severe pain. This result is also consistent with a recent study by Geraghty et al [35] on the use of a DHI in primary care, which aimed to support patients in self-managing their LBP, who found mild and severe pain as barriers to DHI use. Thus, in primary care, DHIs for the self-management of LBP seem to be used primarily by patients with sufficient but not severe pain. In line with previous evidence [28], our findings showed that the use of the portal was facilitated by the high self-motivation of patients.

Barriers to the use of the portal included other more intensive back pain therapies and other acute conditions. Similar to our findings, Geraghty et al [35] described concurrent health conditions and comorbidities as barriers, although these specific comorbidities differed. Mental health conditions, especially depression, which is often associated with LBP [43], were not mentioned in previous studies as a barrier. However, the hindering effect of depression due to reduced drive and low energy has been observed with respect to the use of face-to-face pain self-management programs [44,45]. If depression hinders patient engagement in face-to-face programs, this hindering effect should be even stronger with DHIs such as *tala-med,* which do not involve direct contact with an HCP and thus provide less-direct guidance and encouragement for use. Furthermore, we also found that comorbidities could facilitate patient use of a DHI if this had a positive effect on both LBP and the comorbidity, for instance, in the case of hypertension and LBP. Similar to previous studies [28,29,42,46,47], we also found that technical problems with the portal or little technical skill of the patient were an important barrier to portal use. The technical requirements and required technical skills of a DHI are particularly important because they can exacerbate inequalities in access to quality health information [48,49]. For example, older people and those with lower education and lower income could be disadvantaged, as a larger proportion of these groups do not have access to or cannot use the internet [50,51]. Therefore, when developing and using a DHI, care should be taken to ensure that the information contained could be made available to patients in a nondigital format. The web portal *tala-med* considered this by offering a hard-copy brochure that covered all topics of the portal, as well as printable information graphics, fact sheets, checklists, and exercise sheets that contained summaries of single topics.

Overall, patients perceived *tala-med* as usable, rated it positively, and considered it a clear, comprehensible, trustworthy, and practical resource that they would recommend to others. Our findings are consistent with previous studies showing that approval by an HCP supported patient trust in the quality of the contents of a DHI [29,52]. In addition, they provided insights into further characteristics of the portal content or its presentation that also contributed to the portal’s trustworthiness from the patients’ perspective. The usability of *tala-med* could be further enhanced by offering it as a smartphone app, in addition to greater customization and the inclusion of information to help patients find or contact back pain specialists. The latter 2 suggestions corroborate previous studies in which patients with LBP felt that the DHI could be more customized to their needs and provide an opportunity to contact an HCP [15,53]. Patients perceived *tala-med* as offering added value to other sources of information. In particular, patients felt that they could trust the portal; its contents; and its provision of in-depth, comprehensive, and appropriate information. Trustworthiness, in particular, reflects an important feature of web-based health information [34] that is deficient in many websites [15]. *Tala-med* may remedy the information dilemma faced by many patients when searching for health information on the internet by adding value to self-guided web-based searches.

The practical relevance of *tala-med* and its reported positive effects underline the fact that patients were able to understand and make good use of the portal’s information, while also adding value to the PCP consultation. The ability to use *tala-med* independently and flexibly is an advantage typical of DHIs [28,42]. As a consequence of these features and the incorporated exercise videos, the portal motivates and empowers patients to manage their back pain whenever there is a need or free time to do so. By providing informational, motivational, and practical support, the portal contributed to a decrease in back pain and reduced the need for patients to visit their PCP repeatedly because of LBP. Receiving the portal from their PCP added value to patients. It enriched preceding consultations by allowing patients to repeat and reflect on the contents of the consultation, thus increasing patient informedness while also facilitating and encouraging patient participation in future consultations through increased prior knowledge. These findings are in line with a recent systematic review, which suggests that by using DHIs, “users improved understanding of LBP and enhanced communication with their HCP during subsequent consultations” [28]. Overall, patient perceptions support the utility of the portal, especially when used in combination with PCP consultations.

### Clinical Implications

PCPs could make good use of well-designed web portals for LBP as a supplement to their consultations [35]. Patients welcomed this additional web-based resource if they were familiar with digital technology and the internet. To support patient acceptance of a DHI and its positive effects, it should be integrated into consultations or patient treatment plans. Even if patients can easily use the DHI on their own, PCPs play a crucial role in deciding whether their state of health, in particular pain intensity, comorbidities, and further treatments are compatible with using a given DHI, and which one may offer the most helpful content to patients in terms of prescribing exercises or further information. PCPs and patients benefit from using DHIs such as *tala-med* as a supplement to their consultations [47], as it prepares patients for upcoming consultations and increases their participation. Because the aforementioned implications and their underlying findings are not unique to patients with LBP or *tala-med*, they may be generalized to other DHIs providing health content [21,22] used during and after PCP consultations.

### Strength and Limitations

With 32 half-hour interviews, our qualitative study had a comprehensive information base, including both patients who used *tala-med* and those who did not. Nonetheless, our study relied on a self-selected sample of only 17% of all invited patients. As a result, negative and rare experiences with our portal may have been missed because the patients who experienced them did not participate in our study.

Compared with previous studies, a strength of our study was that patients were able to report their actual experiences with the consultation and the web portal, rather than their anticipated preferences [15]. However, for several patients, these experiences were not particularly vivid at the time of the interview, as the interviews took place at least 4 weeks after the consultation. These patient perceptions of their PCP use of the portal during the consultation may reflect their PCP’s actual behavior unreliably, as subtleties of the consultation, such as brief uses or remarks about the portal during the consultation, may have been forgotten. Recall difficulties may also have led patients to perceive distinguishable aspects of usability less distinctively owing to halo effects [54] and may have led to more socially desirable responses. When investigating details of the portal, such as its usability, it would have been helpful to have the portal open in front of patients, as they were interviewed.

Researchers from different departments developed the portal (AM and SVR) and conducted interviews (CS and M Klimmek). Nevertheless, patients may have mistakenly assumed that they were speaking with someone who had also developed the portal, as the interviewers worked at the same medical center as the portal developers. Thus, patients may have felt inhibited in expressing any criticisms of the portal. However, this limitation only related to patient experiences of the portal. The fact that the interviewers were employed at a university medical center independent of and in a different federal state to that of the PCPs appeared to offer an advantage, in that patients did not have to be concerned when speaking freely about their perceptions of the PCP consultation.

Future studies that quantitatively investigate how patients perceive and evaluate *tala-med* or similar informational DHIs and their effects may mitigate the aforementioned limitations. This could further expand our knowledge of the acceptance, usability, and utility of web portals for LBP.

### Conclusions

Most patients accepted our web portal well. Patient perception also affirmed its usability and utility. *Tala-med* may thus mitigate the information dilemma of patients and seems well suited as a supplement to PCP consultations. The facilitators of and barriers to use in our study are consistent with previous findings and indicate that PCPs should consider pain severity, comorbidities, other therapies, and IT equipment and skills of patients to support their acceptance of the portal. The setting itself, that is, the distribution of the portal by PCPs, seems appropriate as it supports the perceived acceptance and trustworthiness of the portal by patients, as well as bringing added value to current and future consultations. Patient perceptions thus highlight the appropriateness of the portal and the setting. However, they also indicate that many PCPs do not make full use of the portal, rarely integrating it into their consultations. Beyond feedback on *tala-med* and its implementation, the insights of our study on the acceptance, usability, and utility of *tala-med* offer valuable suggestions for the development of DHIs and their successful use as supplements to PCP consultation.

## Appendix

Multimedia Appendix 1 Interview guide.

Multimedia Appendix 2 Allocation of codes to themes.

## Abbreviations

DHI: digital health intervention
GAP: Gut informierte Kommunikation zwischen Arzt und Patient
HCP: health care professional
LBP: lower back pain
OSOP: one sheet of paper
PCP: primary care physician
RCT: randomized controlled trial

## Data Availability

The data sets generated and analyzed during this study are not publicly available because patients were assured in the informed consent form that their personal data would not be made public (according to the General Data Protection Regulation, transcripts of anonymized interviews are considered personal data); however, they are available from the corresponding author upon reasonable request.
