# Supplementary material for: The Acceptance, Usability, and Utility of a Web Portal for Back Pain as Recommended by Primary Care Physicians: Qualitative Interview Study With Patients
Source: JMIR Form Res. 2022 Dec 29;6(12):e38748. doi: 10.2196/38748 (PMC9837709; doi:10.2196/38748)
Supplement: Multimedia Appendix 1 [file formative_v6i12e38748_app1.doc]

## Multimedia Appendix 1. Interview guide.

The following interview guide shows the questions asked of patients who themselves and their PCPs participated in the study in the intended manner. The use of the guide was adaptive, so the order of questions was adjusted as needed to fit the course of the interview. In addition, the guide contained skip rules that served to ensure that interviewees were only asked questions for which they could provide information. It also contained alternative formulations of questions to ensure that patients understood what was being asked, formulations to elaborate on patient responses, as well as additional questions for those who deviated from the intended use of the portal, for example, those who did not use the portal and their reasons why. For simplicity, only the guiding questions were presented in the following guide, without skip rules, alternative formulations, and additional questions about expected deviations from the intended use of the portal.

| Introduction before the interview | | |
| --- | --- | --- |
| Greeting | Greeting and introduction of the interviewer  Thanks for agreeing to participate in this interview | |
| Information phase | Informing the interviewee about the aims of the study   - Project „GAP“: Information offer for patients with back pain - Interviews to learn more about use and usefulness of the GAP offer - Length of interviews: approximately 30 minutes (in one phone call) | |
|  | Information about data protection   - Recording of the interview with a tape recorder to facilitate the evaluation (interviews were transcribed) - Recordings were anonymized (eg, names of interviewee and other persons are made unrecognisable) before analysis - Only the GAP-Team of researchers at Medical Center Freiburg and the University of Freiburg as well as the transcription service have access to the anonymized recordings | |
|  | Any questions before the interview begins? | |
| Important note | Announcement of the tape recording | |
| Start of the interview | | |
| Introduction and interview structure | You and your general practitioner (PCP) had the possibility to use the GAP offer. The GAP offer consists of the information platform *tala-med*, for which you have received the login details from your PCP. We want to know more about how you used and experienced the GAP offer. The interview is in two parts: In the first part, we would like to learn more about the use of the GAP platform during the consultation with your PCP. The second part is about your use of the GAP offer at home. | |
| Part 1: PCP use of *tala-med* during the consultation | | |
| Introduction | You recently visited your PCP because of back pain. | |
| PCP use of *tala-med* during consultation | During the consultation with your PCP: Did your PCP use the information platform *tala-med* to talk to you about your back pain? | |
|  | How did your PCP use the information platform during the interview?   - What did he show to you? Which parts did he use? - Did he recommend specific sections for you to read at home? If so, which ones? How did you feel about them? - Did he print out or send you an information sheet? If so, which one? - Did your PCP explain treatment alternatives to you using the information platform? | |
| Differences of this consultation | What was different about this consultation with your PCP?   - What did he show to you? What parts did he use? Thinking about how a decision was made regarding your treatment: What was different about this compared to previous consultations? | |
| Evaluation of the consultation | Overall, how did you perceive the consultation using the information platform? | |
| Future use of *tala-med* in PCP consultations | Would you like the information platform to be used in future consultations? If yes, how should your PCP use it? | |
| Part 2: Patients use of *tala-med* at home | | |
| Introduction | | You’ve had and have the opportunity to use the GAP offer at home |
| Usage of *tala-med* at home | | Have you already used the information platform?  If the platform was not used: What were your reasons for not using it? |
|  |  | How have you used the information platform so far?   - When was the first time you used it after your visit to the doctor? - How often have you used the platform? - What did you use? (Which sections, which contents?) - Have you used certain content more than once? |
| Ratings of *tala-med* | | How do you rate the information platform overall? |
|  |  | How do you rate the design of the information platform? |
| Usability of *tala-med* | | How did you find your way around the information platform?   - How clear did you find the platform? - If you were looking for specific information, how easy was it for you to find this information? |
| Ratings of the information | | How do you rate the information on the information platform?   - How useful was the information? - Which information did you find most helpful? - How understandable was the information? - How trustworthy did you find the information provided by the information platform? - How did you feel about the amount of information? (Adequacy) - To what extent did the information meet your expectations? - What added value does the information platform have for you in contrast to other sources of information? |
| Whishes | | What else would you like to see from the information platform? |
| Barriers of use | | Were there any external factors that made it difficult for you to use the information platform? |
| Implementation | | What advice from the information platform have you already implemented? |
| Added value to PCP visit | | What additional benefit did the information platform have for you in addition to the PCP visit? |
| Effect of *tala-med* use on following conversations with PCP or HCP | | Have you had further consultations with your PCP or other healthcare professionals (HCPs)? If yes: What was different about these consultations compared to before you used the platform? |
|  |  | Will your use of the portal influence future consultations with your PCP or other healthcare professionals? |
| Recommendation of *tala-med* | | If the portal were freely available, would you recommend it to others? If yes, to whom? |
| Anything to add? | | Anything important that we’ve left out so far, that you would like to add or let us know about? |
| Patient characteristics (questions with predefined answer options as shown in Table 1) | | |
| Sociodemografics | | Gender, age and educational background |
| Back pain | | Do you currently still have back pain?  When did you first experience back pain in your life? |
