# Supplementary material for: The Acceptance, Usability, and Utility of a Web Portal for Back Pain as Recommended by Primary Care Physicians: Qualitative Interview Study With Patients
Source: JMIR Form Res. 2022 Dec 29;6(12):e38748. doi: 10.2196/38748 (PMC9837709; doi:10.2196/38748)
Supplement: Multimedia Appendix 2 [file formative_v6i12e38748_app2.doc]

## *Multimedia Appendix 2*. Allocation of codes to themes.

| Code | Theme |
| --- | --- |
| PCP use of the brochure in the consultation (dichotomous) | PCP use of the portal during the consultation |
| PCP use of the portal in the consultation (dichotomous) |
| PCP use (what was used and how) |
| PCP recommendations to *tala-med* |
| Did PCP offer a printout? |
| Differences of this consultation |
| Overall evaluation of this consultation |
| Future use of *tala-med* in PCP consultations |
| Patient's use of the brochure (dichotomous) | Patient use of the portal |
| Patient's use of the portal (dichotomous) |
| Patient’s first use of the portal |
| Patient's use of the portal (what, how often, why, when) |
| Useful and beneficial (which parts of the portal) |
| Barriers and difficulties |
| Patient's future use of *tala-med* |
| Patient's recommendation of *tala-med* |
| Overall rating of *tala-med* | Usability |
| Ratings of the portal's design |
| Ratings of the portal's clarity and how patients find their way around |
| Ratings of the portal's comprehensibility |
| Ratings of the portal's trustworthiness |
| Patient’s wishes for the portal and suggestions for improvements |
| Added value of *tala-med* to other sources of information | Added value of the portal |
| Added value of *tala-med* to the PCP consultation |
| Effects of *tala-med* use on following conversations with PCP | Effects of the portal |
| Useful and beneficial (why, effects of using the portal) |
